# Increased BDNF may not be associated with cognitive impairment in heroin-dependent patients

**DOI:** 10.1097/MD.0000000000006582

**Published:** 2017-04-14

**Authors:** Xiaoqian Luan, Jingyan Tao, Jie Zhang, Ying Xie, Xiangyang Zhang, Hang Su, Jincai He

**Affiliations:** a Department of Neurology, The First Affiliated Hospital of Wenzhou Medical University, Wenzhou; b Department of Rehabilitation Medicine, Sir Run Run Shaw Hospital, College of Medicine, Zhejiang University, Hangzhou; c Shanghai Mental Health Center, Shanghai Jiaotong University School of Medicine, Shanghai; d Beijing HuiLongGuan Hospital, Peking University, Beijing, PR China; e Menninger Department of Psychiatry and Behavioral Sciences, Baylor College of Medicine, Houston, TX, USA.

**Keywords:** brain-derived neurotrophic factor, cognition, heroin, impairment

## Abstract

A growing number of evidence suggests that brain-derived neurotrophic factor (BDNF) plays an important part in modulating the activities on the basis of hippocampus neural plasticity, such as learning and memory. Heroin addiction has a series of cognitive impairments that may be associated with BDNF. In this study, we explored the association of BDNF with cognitive function in heroin-dependent patients.

We enrolled 86 heroin-dependent patients and 238 normal control subjects and examined their cognition by the repeatable battery for the assessment of neuropsychological status (RBANS) and serum BDNF levels in 2 groups.

BDNF levels were significantly higher in patients than controls (*P* < .001). Cognitive scores of the RBANS showed that attention and language index (*P* < .05) were significantly lower in heroin-dependent patients than control groups. Unfortunately, we found no positive association between BDNF and cognitive function in patients, except that BDNF was positively associated with visuospatial/constructional index in control groups.

Our findings suggest that BDNF may not be involved in the pathophysiology of heroin dependence, but more studies about cognitive impairment in heroin addiction are needed.

## 1 Introduction

Cognitive impairment is a typical feature of the long-term use of heroin, including learning, memory, and executive function deficits.^[1,2]^ These effects will aggravate the burden of rehabilitation in heroin-dependent patients and reduce their prognosis to a great degree. However, the pathophysiological mechanisms underlying these cognitive deficits associated with long-term heroin use are still not well understood. Therefore, investigating neurobiological changes in heroin dependent individuals has great potential to improve the understanding of the disease and help to improve their daily life function.

Brain-derived neurotrophic factor (BDNF) is one of the neurotrophin family of growth factors, plays an important role in learning and memory, and modulates synaptic plasticity of the midbrain dopaminergic and cholinergic neurons.^[3,4]^ Animal studies revealed that inhibiting BDNF signaling would influence cognitive functions such as learning and memory, and possible mechanisms may be associated with long-term potentiation (LTP) induced by BDNF in dopaminergic neurons.^[5,6]^ Moreover, it was found that BDNF expression changed in the VTA in chronic opiate administration animal models.^[7]^ Furthermore, our previous research has reported a significant increase of serum BDNF levels in heroin-dependent patients compared to healthy controls.^[8]^

In the view of cognitive impairments and the marked changes in serum BDNF existed in heroin-dependent patients, and the important implication of BDNF in cognitive function, it would be of interest to explore the association between cognitive deficits and serum BDNF levels in heroin addiction. Therefore, the purpose of this study was designed to determine the relationships of serum BDNF levels with cognitive function in heroin-dependent patients and healthy groups.

## 2 Methods

### 2.1 Subjects and setting

Eighty-six Chinese heroin-dependent patients were recruited from Wenzhou Sanyang Detoxification Institute, which is located in the south of Zhejiang province, China. All patients met the Diagnostic and Statistical Manual of Mental Disorders, 4th edition (DSM-IV) criteria for heroin dependence as diagnosed by 1 clinical psychiatrist. The inclusion criteria: age 18 years or older, positive urine test for opiates upon admission, have no current medical treatment and any other medication for withdrawal, and heroin abstinence for 1 to 7 days (period between enrollment and last drug use). The exclusion criteria: seropositivity for HIV, have serious medical illnesses that required pharmacological treatment, have neurological disease, have acute withdrawal symptoms, or met the DSM-IV criteria for axis I psychiatric disorder or drug dependence other than heroin and nicotine.

The 238 control groups were recruited from the physical examination center at the First Affiliated Hospital of Wenzhou Medical University. All controls had no current or histories for substance dependence or abuse and psychiatric illnesses, no neurological disease, and no family histories for psychiatric illnesses.

All subjects gave written consent with admission for the use of their clinical data and their blood samples for research purposes, which was approved by the institutional review board and the ethics committee of The First Affiliated Hospital of Wenzhou Medical University. Both the patients and controls are Han Chinese according to their identification card.

### 2.2 Measures

A case report form including sociodemographic characteristics (sex, age, education, weight, height, etc.), drug use history (total duration of heroin use, age of onset, etc.), cigarette smoking, and alcohol drinking was administrated to each subject by one psychiatrist in a separate room. In controls, we only got demographic characteristics (sex, age, education, weight, and height).

### 2.3 Serum brain-derived neurotrophic factor measurement

Serum samples from healthy controls and patients were collected between 8 and 10 am at the same period. Five milliliter of blood was obtained and centrifuged at 3500 rpm for 10 minutes immediately. Serum was separated and stored at −80° before use. Serum BDNF levels were measured using DuoSet ELISA Development System (Catalog number DY248, R&D Systems, America). All measurements were conducted by trained operators blind to the research design according to the manufacturer's instruction. All assays were performed in duplicate and expressed as pg/mL. The detection range of this assay was 20 to 4000 pg/mL. The intra- and interassay coefficients were <5% and <10%, respectively.

### 2.4 Assessment of the repeatable battery for the neuropsychological status (RBANS, Form A)

The RBANS (Form A) was administered to measure cognitive function by trained psychiatrists who had got assessing qualifications at the same time with the case report form interview. The RBANS consists of 12 subtests that are used to calculate 5 age-adjusted index scores and a total score. Test indices include immediate memory (list learning and story memory tasks), visuospatial/constructional (figure copy and line orientation tasks), language (picture naming and semantic fluency tasks), attention (digit span and coding tasks), and delayed memory (list recall, story recall, figure recall, and list recognition tasks). The RBANS was previously translated into Chinese, and its clinical validity and test–retest reliability were established.^[9]^

### 2.5 Statistical analysis

Demographic and clinical variables of the patient and control groups were compared using *t* test, analysis of variance (ANOVA) for continuous variables, and chi-squared for categorical variables. Since the BDNF variables were normally distributed in patients and normal controls (Kolmogorov–Smirnov one-sample test; both *P* > .05), the principal outcome analysis consisted of one-way ANOVA. Where there was significance in ANOVA, the effect of sex, age, education, and body mass index (BMI) was tested by adding these variables to the analysis model as covariates. Relationships between variables have been assessed with Pearson product moment correlation coefficients. Bonferroni corrections were applied to each test to adjust for multiple testing. Single regression analysis was used to assess the correlations between cognitive function shown on the RBANS total score and its index scores and BDNF. The degree of association among independent variables, including age, sex, education years, BMI in both patient and controls, and clinical variables in patient group, such as age of onset, total duration of heroin use, were examined by multivariate regression analyses (stepwise regression model). SPSS version 16.0 was used to do all statistical analyses. Statistical significance was defined as *P* < .05.

## 3 Results

### 3.1 Characteristics of heroin-dependent patients and control groups

Clinical demographic characteristics of heroin-dependent patients and control groups are presented in Table 1. There were significant differences in age, education, and BMI (all *P* < .001) between patients and controls, which were adjusted in the following analyses.

**Table 1:**
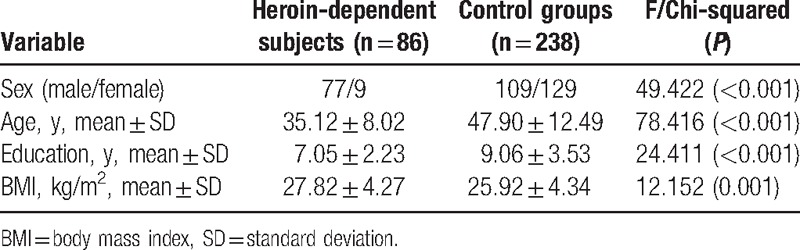
Demographic characteristics in control groups and heroin-dependent patients.

There was no significant association between BDNF levels and gender, age, education, and BMI in control groups (all *P* > .05). On the other hand, in patient group, BDNF was found to be associated with age (*r* = 0.318, *P* = .005). In addition, we noted a significant correlation between serum BDNF levels and onset age of heroin use (*r* = 0.315, *P* = .006), while no association with alcohol use, nicotine use, and total duration of heroin use.

### 3.2 BDNF levels between patients and controls

Serum BDNF levels were markedly higher in patients than in controls (1692.94 ± 707.71 vs 1194.46 ± 230.98 pg/mL, F = 89.496, df = 1315, *P* < .001), which was presented in Table 2. When the effect of age, education, and BMI was examined by adding them to the ANOVA as covariates, a significant difference between patients and controls was still observed (F = 60.979, df = 6309, *P* < .001).

**Table 2:**
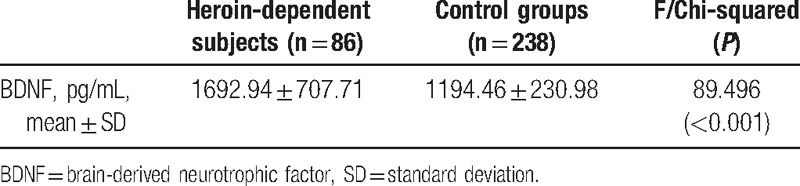
Brain-derived neurotrophic factor levels between control groups and heroin-dependent patients.

### 3.3 Cognitive scores of the RBANS between heroin-dependent patients and control groups

RBANS total and index scores of 86 patients and 238 control groups are shown in Table 3. There were significantly lower cognitive scores on attention and language index (*P* < .05) but no significant on any other index scores or the RBANS total score (*P* > .05) in heroin-dependent patients than normal controls. These significant differences were still significant when adjusting for age, education, and BMI (all *P* < .05; Table 3).

**Table 3:**
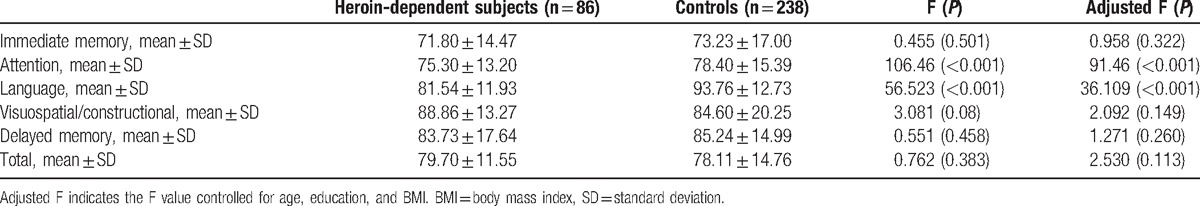
Comparisons of total and index scores on the repeatable battery for the assessment of neuropsychological status by 2 groups.

### 3.4 The relationships between cognitive scores of the RBANS and serum BDNF levels

For the control groups, correlation analyses showed a significant positive association between BDNF and visuospatial/constructional index (*r* = 0.146, df = 230, *P* = .026), for the patients, single regression analysis showed that BDNF was not associated with any index and total scores of RBANS.

Multiple regression analysis was performed to elucidate independent determinants of RBANS and its index scores. For the control groups, BDNF was not found to be a contributor to any of the RBANS index or its total score (all *P* > .05) except for the factor of education year (*P* < .001). For the patients, BDNF was not found to be contributors to any RBANS index and total score.

## 4 Discussion

The main findings of this study were BDNF levels were significantly higher in heroin-dependent patients during early withdrawal in our previous research, significantly lower cognitive scores on the RBANS attention and language score but no difference on other subscales or total score were found in patients than normal controls, and BDNF was not associated with cognitive function in heroin-dependent patients or control groups.

In this study, we found increased serum levels of BDNF in heroin-dependent patients during early withdrawal period, which has been discussed in our previous study and is consistent with others but not all.^[8,10,11]^ BDNF has been mentioned in the pathophysiology of opiate dependence. Change of BDNF levels have been reported in heroin addicts and withdrawal. Heberlein et al^[11]^ have found positive association between opiate craving and the serum level of BDNF in opiate-dependent patients, which could cause the relapse and addiction of heroin. Meanwhile, our previous studies have investigated increased serum BDNF levels during the early withdrawal in heroin or methamphetamine dependence, and serum BDNF levels were positively related to psychotic symptoms such as depressive symptoms and impulsivity.^[8,12]^ Also, some researchers have shown that promoter methylation of BDNF was associated with drug dependence such as heroin and alcohol.^[13]^ Thus, it can be seen that BDNF played a significant role in heroin dependence. In addition, the role of BDNF in addiction involved a series of mechanism. In rat, cocaine causes learning and memory impairments involve the mechanism of oxidative stress, which could participate in drug addiction and drug toxicity.^[14,15]^ Recent researches have investigated that BDNF and oxidative stress markers thiobarbituric acid reactive substances took participation in heroin addiction include cerebral plasticity and impairment, which could reflect the severity of drug abuse.^[16]^ Therefore, it was not only that BDNF but also oxidative stress markers played the role in addiction. Also, the role of extracellular signal regulated kinase cascade alteration induced by BDNF^[17–19]^ and the role of AMPA receptors in synaptic transmission in the central nervous system played in drug addiction include heroin.^[20–22]^ Drug addiction referred to complicated mechanism, we need further exploration about addiction in the future.

Heroin-dependent patients had significantly lower cognitive performance on the attention and language score but not other subscales or total RBANS score than normal controls. These results are consistent with the majority of studies assessing cognitive performance in heroin addiction to some degree, suggesting that heroin indeed impair cognitive function, which influence their daily function further. Neuropsychological reviews indicated acute and chronic opioid users had deficits in attention, concentration, memory recall, visuospatial function, spatial working memory, psychomotor speed, and executive functions, which buprenorphine (a partial mu opioid agonist) or methadone maintenance treatment could improve these cognitive impairments to a certain extent.^[12]^ In this study, we only found cognitive deficits on attentional and language functions in heroin users but not on visuospatial or memory. The possible reason of this inconsistence may be due to different stages of disease progression (still using heroin or withdrawal), differences in total duration of heroin use.

The current findings indicate that serum BDNF levels were not associated with cognitive functions no matter in patients or controls. This finding was similar to a recent research, which suggested that positive association was existed between smoking severity and serum BDNF levels and smoking caused decline of cognition; however, cognitive decline was not associated with serum BDNF levels.^[23]^ One study about preschool children has found that plasma and serum BDNF levels were negatively related to cognition.^[24]^ In addition, an animal experiment showed that nicotine dependence can give rise to increased BDNF levels of striatal and prefrontal, but made cognitive decline.^[25]^ Our finding is not consistent with 2 recent studies showing that higher serum BDNF levels were associated with better neuropsychological function in healthy older adults, especially in aging women.^[26,27]^ Preclinical researches hypothesized that BDNF could regulate synaptic plasticity through the induction of long-term potentiation (LTP), one of the main molecular mechanism which form the basis of learning and memory.^[5,6]^ The above conclusions have shown that the relationship between BDNF levels and cognition existed difference. Except for the significant role of BDNF played in cognition. There were also a lot of neurochemical reactions that can alter the cholinergic, glutaminergic, and catecholaminergic system to affect cognition. In mice, researchers have illustrated that BDNF signaling balanced dopamine-glutamate of striatal to regulate cognitive control processes.^[28,29]^ Cognitive impairment is associated with altered synaptic plasticity and enhanced hippocampal glutaminergic expression.^[30]^ In rats, cholinergic and neurotrophin markers, which took participate in spatial learning ability, affected cognitive decline and improvement.^[31,32]^ We only explored association between BDNF levels and cognition, but lacked the research about the role of intermediated products in cognition. Numerous factors may have contributed to these inconsistence such as differences in techniques of measuring BDNF levels, different research material (serum or plasma), sampling of patients in different stages of disease progression (still using heroin or withdrawal), diverse treatment, or the biological heterogeneity (physiology differences) may be responsible for the discrepancy. This study presented other confusing effects such as education years on BDNF levels, which needs to be further explored.

Although BDNF is highly present in the nervous system, it is also highly concentrated in peripheral blood serum of humans and rats, and a significant correlation between serum BDNF and central BDNF has been reported.^[33,34]^ However, it is unknown whether changes in serum BDNF levels come from alterations of BDNF levels in the periphery, brain, or both. In the periphery platelet is the main place to combine, store, and release of BDNF, but in the central, neurons is the main place to produce BDNF, such as hippocampal neuron.^[35–38]^ Therefore, what extent peripheral BDNF levels could reflect BDNF in the brain still remains unclear. We did not found a relationship between serum BDNF and cognition, but we could not reach a conclusion that central BDNF was not associated with cognitive function. Further studies measuring BDNF in nervous system are needed.

Furthermore, we found that age had some effect on serum BDNF levels in the heroin group. The older the age of heroin-dependent patients, the higher the BDNF serum levels. Several lines of work are consistent with our finding. For example, an age-dependent increase in serum BDNF levels has been reported for women.^[39,40]^

Several limitations should be mentioned in this study. First of all, as noted above, we measured BDNF levels in the serum, not in the brain, which may be the main reason that the results were negative. Second, we did not perform MRI to the participants. Moreover, BDNF regulates drug-induced behavior in a very complex manner that varies from one brain region to another, which needs more deep technology of brain imaging that we could not achieve. In the future, we will explore this field as much as possible. The present study was just a tentative exploration, which was aimed to seek common character. Third, this study is a small sample size and cross-sectional design, we will increase the sample size and make further efforts to follow up the patients. In addition, psychological factors such as social environment stress could also affect the determination of BDNF levels and cognitive function.

In summary, BDNF increased in heroin-dependent patients during early withdrawal compared with control groups. Heroin-dependent patients showed cognitive impairments in attention and language function. Meanwhile, increased BDNF may not be associated with cognitive impairment. Further study is necessary to elucidate the role of central BDNF in opiate addiction and cognitive function. We will focus the future research on fundamental experiment study and translational research from basic to clinical. It is not clear that a series of complicated mechanism from cell and molecular level to protein expression of BDNF. To deeply understand the role of BDNF in addiction, we need embark on mechanism research from genetics, molecule, and cell to entirety and association research from endophenotype to phenotypes.

## Acknowledgments

The authors thank the National Key Technology R&D Program in the 11th Five Year Plan of China (2009BAI77B06) and National Key Technology Research and Development Program of the Ministry of Science and Technology of China (grant number: 2015BAI13B01) for the support. The authors also thank the staff and the patients with stroke for their contributions during this study.
